# Navigating the pause—patient experiences with perioperative anticoagulation interruption: a qualitative study

**DOI:** 10.1016/j.rpth.2026.106890

**Published:** 2026-07-28

**Authors:** Alfonso Tafur, Sally Jensen, Albert Marquez, Steven Greenberg, Barry Wallach, Jean Connors, Jerrold Levy, Marisa Durante, Alexis Preston, James Douketis

**Affiliations:** 1 Vascular Medicine, Endeavor Health, Evanston, Illinois, USA; 2 University of Chicago, Pritzker School of Medicine, Chicago, Illinois, USA; 3 Research Institute, Endeavor Health, Evanston, Illinois, USA; 4 Mayo Clinic, Rochester, Minnesota, USA; 5 Department of Anesthesia, Endeavor Health, Evanston, Illinois, USA; 6 Patient Representative, Illinois, USA; 7 Brigham and Women’s Hospital, Boston, Massachusetts, USA; 8 Duke University Medical Center, Durham, North Carolina, USA; 9 Cardiovascular Institute, Endeavor Health, Evanston, Illinois, USA; 10 St. Joseph’s Healthcare Hamilton, McMaster University, Hamilton, Ontario, Canada

**Keywords:** anticoagulants, focus groups, patient experience, perioperative care, qualitative research

## Abstract

**Background:**

Patients on chronic anticoagulation frequently undergo temporary medication interruption for surgical or invasive procedures, yet the patient perspective on this experience remains poorly characterized.

**Objectives:**

The aim of this study was to identify the concerns, preferences, and unmet needs of patients undergoing perioperative anticoagulation interruption to inform patient-centered care strategies.

**Methods:**

A qualitative study using 7 semistructured focus groups was conducted between 2022 and 2023 at a single academic health system. Twenty-two adults on chronic oral anticoagulation who had recently undergone planned interruption for high-risk procedures were enrolled. Groups were stratified by anticoagulant type (warfarin vs direct oral anticoagulant [DOAC]). Emergent themes from focus group transcripts were identified using grounded theory with iterative open coding and consensus analysis via Dedoose software.

**Results:**

Nineteen themes were identified and consolidated into 5 core categories: (1) Risk Perception, reflecting fears of bleeding and clotting shaped by personal history; (2) Communication and Instruction Clarity, emphasizing the need for clear, timely guidance; (3) Self-Management and Logistical Challenges, including difficulties with injections, medication tracking, and dietary management; (4) Healthcare System Gaps, including care coordination failures and access delays; and (5) Emotional and Adaptive Responses, including stress and creative coping strategies. Bruising and injection challenges were more prominent concerns than major clinical events.

**Conclusion:**

Perioperative anticoagulation interruption poses substantial informational, emotional, and logistical burdens on patients. Enhancing communication, supporting self-management, and improving system coordination are essential components of anticoagulation stewardship. Integration of patient-reported outcomes into future trials is warranted.

## Essentials

• Millions stop blood thinners before surgery each year.
• We interviewed 22 patients in focus groups after planned treatment interruption.
• Patients struggled more with confusion and injections than major complications.
• Clear instructions and better teamwork could make care safer.

## 1 Introduction

Systems of care aiming to improve outcomes and accountability must involve all stakeholders. Patients receiving chronic anticoagulation for atrial fibrillation (AF) or venous thromboembolism often require temporary anticoagulant interruption for surgical or invasive procedures. Such interruptions occur in an estimated 15% to 20% of these patients annually, necessitating careful perioperative anticoagulant management to mitigate clinically important adverse outcomes [[1], [2], [3]].

Fortunately, evidence-based guidance exists for both warfarin-treated and direct oral anticoagulant (DOAC)-treated patients [[4], [5], [6], [7], [8]]. However, the perioperative period also poses significant clinical and emotional challenges, as patients must navigate the tension between bleeding and thrombotic risks while adhering to complex medical instructions. Although clinical guidelines provide evidence-based strategies, the patient experience—particularly patient understanding, concerns, and informational needs—remains poorly defined [9].

Previous studies have highlighted variability in provider practices and system-level workflows in anticoagulation management [10]. However, limited qualitative data capture the voice of patients directly impacted by these decisions—particularly those with chronic conditions requiring long-term anticoagulation. Understanding how patients perceive perioperative instructions, what information they find lacking, and what support systems they rely upon is essential for designing patient-centered interventions. Perioperative anticoagulation interruption carries competing risks: in a pivotal randomized trial of warfarin-treated AF patients, low-molecular-weight heparin bridging was associated with major bleeding in 3.2% of patients without a reduction in arterial thromboembolism (0.3%) compared with no bridging (1.3% and 0.4%, respectively); similarly, a no-bridging DOAC interruption strategy yielded 30-day major bleeding rates <2% and arterial thromboembolism rates <0.6% [4,5].

This study used focus groups to explore the lived experiences of anticoagulated patients who have recently undergone perioperative interruption. The objective was to define the needs, preferences, and concerns of patients undergoing perioperative anticoagulation interruption through structured focus group discussions, with the goal of improving clarity, safety, and patient-centeredness of the perioperative anticoagulation process.

## 2 Methods

### 2.1 Study design

This qualitative study employed a focus group design to explore patient-centered concerns triggered by episodes of perioperative anticoagulation management. The study was approved by the institutional review board; all participants provided written informed consent. The study was conducted in accordance with the Consolidated Criteria for Reporting Qualitative Research (COREQ) [11].

### 2.2 Participants and recruitment

Eligible unrelated participants were adults on chronic oral anticoagulation for any indication who had experienced a planned interruption of therapy within 6 months of enrollment. Only patients whose anticoagulant interruption was for a major-risk procedure, as defined in the BRIDGE trial [5], were recruited. A convenience sample of subjects were contacted from anticoagulation clinic logs, the principal investigator’s clinical practice, and surgical case logs. Consenting research coordinators emphasized confidentiality including discretion among fellow participants and consent for digital recording and that the main goal of the study was to evaluate the perioperative anticoagulation process. Shared decision making regarding bridging anticoagulation was standard practice and prerequisite for inclusion. An antithrombotic stewardship program is not in place. Participation was voluntary and no compensation was provided.

### 2.3 Focus group procedures

Focus groups were held in person or virtually based on participant preference and availability. Groups were limited to 3 to 5 participants and conducted approximately every 4 to 6 weeks to allow classification of emerging themes. The interview format was semistructured, guided by open-ended questions developed by a multidisciplinary panel. The anchoring question asked patients to reflect on their concerns during the process of stopping anticoagulation before a procedure; sample questions targeted emotional, physical, and lifestyle dimensions (see Supplement).

Each focus group lasted 45 to 75 minutes. The lead author (A.T., male, physician) served as facilitator for all sessions; research collaborators (A.P., female, research coordinator; and M.D., female, research coordinator) maintained field notes capturing nonverbal cues and group dynamics. All researchers involved in the conduct of focus groups were trained in qualitative research methodology. Sessions were video and audio recorded, transcribed verbatim, and anonymized. In several cases, participants’ spouses made comments virtually; however, spouses were not recruited as participants. Additional demographic and clinical data were abstracted following consent. Videos were deleted after the transcript summary with nonverbal cues was finalized. Transcripts were not returned to participants for comment and/or correction. Patients were grouped by anticoagulant agent (warfarin vs DOAC) after the first 2 mixed sessions to facilitate within-group homogeneity.

### 2.4 Data analysis

Data collection and analysis proceeded concurrently to allow iterative refinement of the interview guide and determination of thematic saturation, defined as the point at which no new themes emerged. Two investigators (A.T. and S.J.) independently performed open coding using *in vivo* coding to preserve participant language. Coding was conducted using Dedoose software (version 9.0.107 [2023]; SocioCultural Research Consultants, LLC). Regular consensus meetings were held to review codes using grounded theory and integrate feedback before proceeding to additional focus groups. Once saturation was reached, investigators summarized, clustered, and selected exemplar quotes.

A subgroup comparison of theme frequency was conducted using qualitative comparison of endorsement counts between DOAC and warfarin participants. Participants did not provide feedback on the findings.

## 3 Results

### 3.1 Participant characteristics

Of 95 patients contacted, 22 (23%) participated and 73 (77%) declined. Among nonparticipants, 36 did not respond to outreach, 12 declined due to being busy, 6 cited work obligations, 11 declined without reason, 2 were unable to use videoconferencing, 5 expressed initial interest but did not respond to follow-up, and 1 did not attend.

Twenty-two adults currently prescribed anticoagulants participated in 7 focus groups (5 in person, 2 virtual). After the first 2 combined sessions, subsequent groups were DOAC only (*n* = 2 groups) or warfarin only (*n* = 3 groups). The mean number of participants per group was 3.1 (range, 3-4). All but 3 patients received enoxaparin at some moment in the interruption process. No participants reported a major bleeding or clotting complication during the index perioperative period. Participant characteristics are summarized in Table 1.

**Table 1:** Sociodemographic and clinical characteristics of focus group participants (*N* = 22).

Variable	*n* (%)
Sex, male	13 (59.1)
Age, median (range), y	68 (41-69)
Education	
High school diploma/GED	3 (13.6)
Some college/associate degree	3 (13.6)
College degree	12 (54.5)
Advanced degree	4 (18.2)
Race	
White	19 (86.4)
Black/African American	3 (13.6)
Employment status	
Employed full-time	3 (13.6)
Work from home	2 (9.1)
Retired	15 (68.2)
On disability/leave of absence	1 (4.5)
Current anticoagulant type	
Direct oral anticoagulant	10 (45.5)
Warfarin	12 (54.5)
Indication for anticoagulation	
History of venous thromboembolism	11 (50.0)
Atrial fibrillation	7 (31.8)
Mechanical heart valve	2 (9.1)
Other	2 (9.1)

### 3.2 Thematic findings

Participants discussed a wide array of themes related to perioperative anticoagulant interruption, including concerns, symptom experience, patient-provider interaction, perceptions of anticoagulants, and management strategies. Nineteen key themes emerged across focus groups. Table 2 displays the frequency of participant endorsement, and Table 3 maps initial themes to 5 consolidated core categories. Representative quotations for each theme are presented in the Figure and Table 3.

**Table 2:** Focus group participant endorsement of key themes (*N* = 22).

Perioperative anticoagulation interruption theme	Total (*N* = 22)	DOAC (*n* = 10)	Warfarin *(n* = 12)
Value of written instructions	18 (81.8)	8 (80.0)	10 (83.3)
Injections (enoxaparin)	16 (72.7)	6 (60.0)	10 (83.3)
Access to team	13 (59.1)	7 (70.0)	6 (50.0)
Clotting risk	13 (59.1)	6 (60.0)	7 (58.3)
Satisfaction with communication	13 (59.1)	3 (30.0)	10 (83.3)
Provider trust	12 (54.5)	3 (30.0)	9 (75.0)
Clear scheduling guidance	11 (50.0)	6 (60.0)	5 (41.7)
Tracking via calendar tools	11 (50.0)	4 (40.0)	7 (58.3)
Bruising awareness	10 (45.5)	4 (40.0)	6 (50.0)
Fear of excessive bleeding	10 (45.5)	4 (40.0)	6 (50.0)
Reliance on personal tracking tools	9 (40.9)	5 (50.0)	4 (33.3)
Reliance on partner	9 (40.9)	3 (30.0)	6 (50.0)
Food concerns	8 (36.4)	0 (0.0)	8 (66.7)
Perioperative bleeding fear (INR-related)	8 (36.4)	0 (0.0)	8 (66.7)
Access delay	6 (27.3)	3 (30.0)	3 (25.0)
Confidence in anticoagulation	6 (27.3)	3 (30.0)	3 (25.0)
Medication contrast with warfarin	5 (22.7)	2 (20.0)	3 (25.0)
Medication cross-check reassurance	5 (22.7)	3 (30.0)	2 (16.7)
Technology tools	5 (22.7)	1 (10.0)	4 (33.3)

Values are *n* (%).

DOAC, direct oral anticoagulant; INR, international normalized ratio.

**Table 3:** Mapping of initial themes to core categories.

Core category	Corresponding initial themes	Exemplary quotations
1. Risk Perception	Clotting risk; bruising awareness; perioperative bleeding fear; medication contrast with warfarin	*“My only concerns was being off the [apixaban] Eliquis, how soon a blood clot could form since I had two pulmonary embolisms. That was my only concern.” (002, DOAC)* *“I didn’t expect the bruising. So I wasn’t warned of it as symptoms or something, or maybe I didn’t, I glanced over that.” (011, DOAC)* *“Because before I would have to, uh, go off the coumadin for like…five days. And then I’d have to go back on right after the procedure. And there was a fear there.” (001, warfarin)*
2. Communication and Instruction Clarity	Value of written instructions; satisfaction with communication; clear scheduling guidance; provider trust; medication cross-check reassurance	*“I prefer the, from the doctor, physicians, whoever, explain it verbally to me face to face, then I get the notes that I can reference, because if I don’t remember something or I get something mixed up, the notes are right there to just flash it back to my head.” (083, warfarin)* *“I’ve been pretty satisfied with how it’s been explained to me. And then the follow up instructions on print, um, they’ve always been, I always had somebody accessible if there’s any other questions. So at this point I have nothing I can say that I would look to to improve any of that…” (083, warfarin)* *“…I merely had to quit taking the medication on a particular day, and commence on a separate day, on a later day, yeah, it was a simple process for me, and the communication was straightforward.” (012, DOAC)* *“The surgeon, but when I objected to not receiving the extra blood thinner, he said, well, that was the cardiologist’s decision. And I didn’t even know he was involved other than a pre-exam before the surgery.” (082, warfarin)* *“…two calls preoperative from the hospital. The first call was a week before my surgery, and she went through every one of my medications and when I should continue or discontinue those. And then the person that called the day before the surgery went through it again. So I felt very comfortable that we were all on the same page.” (006, DOAC)*
3. Self-Management and Logistical Challenges	Injections (enoxaparin); reliance on personal tracking tools; food concerns (self-monitoring aspect); tracking via calendar tools; medication contrast with warfarin (logistics-related)	*“…I didn’t know how to do, I didn’t know how to do the shots myself, I did have somebody else to do them because I couldn't do it myself, but that’s the only issue.” (007, DOAC)* *“…on my iPhone calendar stating, you know, Tuesday night, stop medication.” (011, DOAC)* *“…I would transfer that to my personal calendar so I’m not off track…I would get notifications that way so that I’m not off track. And the notifications are like reminders.” (001, warfarin)* *"Well, [apixaban] Eliquis was just an easier drug to deal with, whereas with the warfarin, I have to go into the clinic, the Coumadin clinic more often. And it’s basically because I’ve never really been in the therapeutic range that long because of the fact that my system does not…um…well my system does not absorb that well.” (001, warfarin)*
4. Healthcare System Gaps	Access to team; access delay; satisfaction with communication (when negative); reliance on personal tracking tools (as adaptation)	*“Very easy to call the office to talk to is the nurse that handled everything and his office assistant. And it was, I was very, very impressed.” (005, DOAC) [Context: also dealing with COVID-19 and MRSA]* *“In fact, that’s one thing I think could be corrected. If you have a patient that’s 3.3 or above an INR, and the nurses want you to go in for a blood test to verify or confirm the reading, that patient should have immediate access to the lab to have the blood drawn and not sit there and wait for two hours till an opening occurs.” (087, warfarin)*
5. Emotional and Adaptive Responses	Reliance on partner; technology tools; fear of excessive bleeding; confidence in anticoagulation (coping aspect)	*“No, no, it’s it, as I said, it’s the fact that this whole thing is invisible to me. Right? And you guys have all kinds of things you can use to look and see and test, but it’s invisible to me. And yeah, at the end of the day, I just have to have faith that the medication is doing what it’s supposed to do. Because I yeah, it’s not like, you know, an injury that you can see and you can monitor it’s just in your body.” (012, DOAC)*

DOAC, direct oral anticoagulant; INR, international normalized ratio; MRSA, methicillin-resistant *Staphylococcus aureus*.

**Figure:**
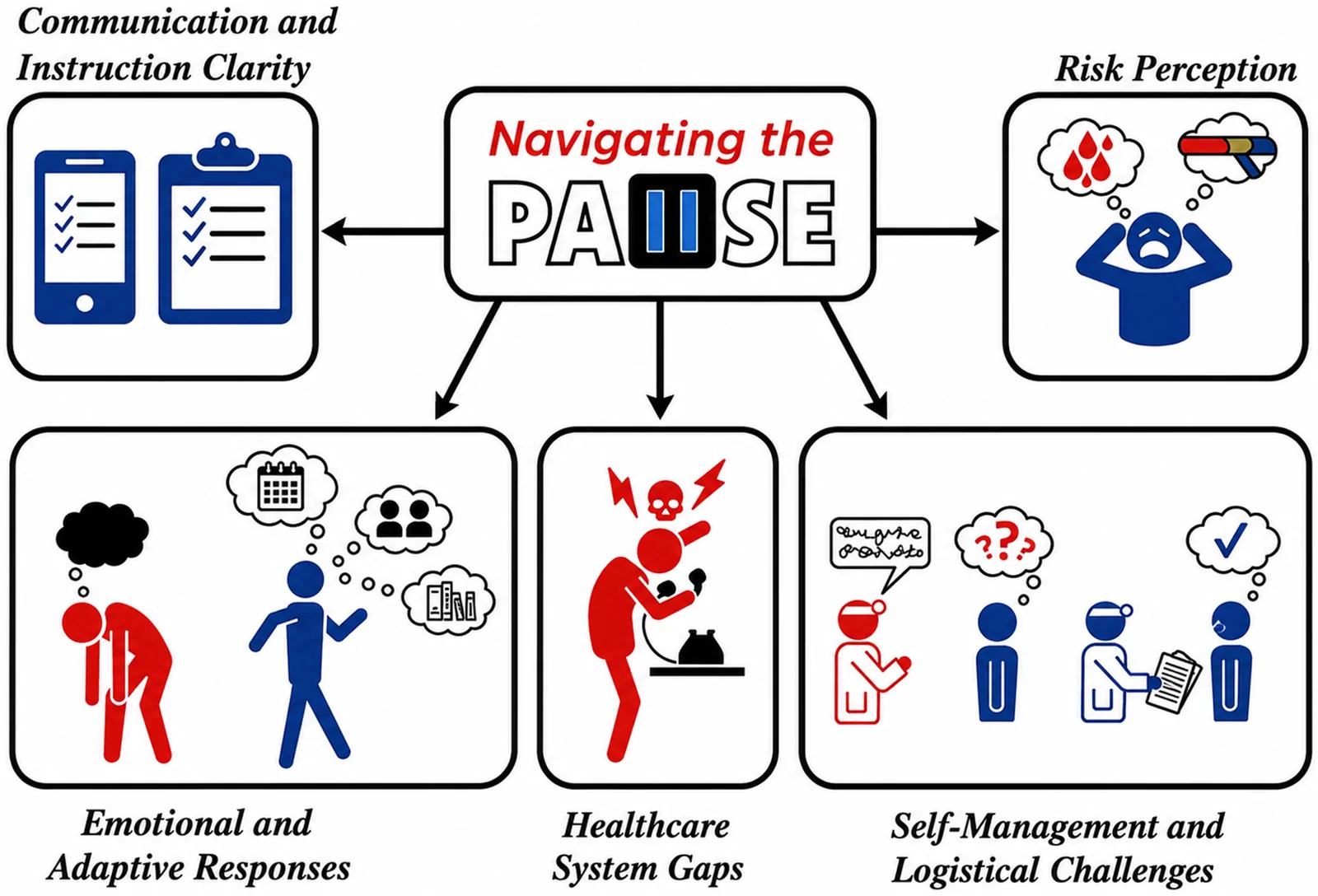
Navigating the PAUSE: Patient-Level and Health-System Factors Influencing Perioperative Anticoagulation Management. Conceptual framework illustrating the interacting factors that may influence patients’ understanding and execution of perioperative anticoagulation instructions. Five interconnected domains are represented: communication and instruction clarity, risk perception, emotional and adaptive responses, healthcare-system gaps, and self-management and logistical challenges. Together, these factors may affect adherence to preprocedural anticoagulant interruption and postprocedural resumption instructions and contribute to variability in the patient experience during perioperative anticoagulation management. PAUSE, Perioperative Anticoagulant Use for Surgery Evaluation.

#### 3.2.1 Risk perception

Patients expressed significant fears about bleeding and clotting, shaped by past events such as pulmonary embolism, stroke, or bleeding complications. Some found reassurance in provider guidance or bridging protocols, whereas others remained uncertain. One participant stated: “My biggest thing is how long do you go before you say, Hey, I need to call the doctor… I was double questioning whether I should go or not. And it turned out I should have went.” (Patient 007, DOAC).

#### 3.2.2 Communication and instruction clarity

Clear, step-by-step instructions—both verbal and written—were essential for reducing anxiety. Most participants (18 of 22; 82%) valued written instructions for reference after verbal communication. Patients valued trusted relationships but noted gaps, particularly around international normalized ratio levels and dietary advice with warfarin. One participant stated: “Here’s what we talked about. Here it is in writing so you have it and you can refer back to it.” (Patient 012, DOAC).

#### 3.2.3 Self-management and logistical challenges

Patients faced difficulties managing injectable anticoagulants (enoxaparin), tracking medications, handling needle disposal, and adjusting for food-drug interactions. Calendars, pillboxes, and written tools were commonly used for support. Sixteen participants (73%) endorsed injection-related challenges, spanning pain, bruising, technique, and needle disposal. No DOAC patients reported food concerns, whereas 8 warfarin patients (67%) did.

#### 3.2.4 Healthcare system gaps

Coordination challenges were prominent: multiteam communication gaps, limited provider availability, and access delays were sources of added stress. Patients frequently assumed responsibility for facilitating communication across surgical, primary care, and anticoagulation teams. One participant noted: “It’s a little bit of a headache having to physically go in and see each of those doctors.” (Patient 002, DOAC).

#### 3.2.5 Emotional and adaptive responses

Despite stress, many patients adapted effectively using technology (smart devices, artificial intelligence tools, patient portals), partner support, and personal tracking strategies. One participant described consulting multiple artificial intelligence platforms to interpret medical results: “I use Claude, I use Gemini… I compare the answers from multiple platforms.” (Patient 084, warfarin) These tools helped patients feel more in control during a vulnerable care transition.

Notably, major outcomes such as overt major bleeding or stroke were infrequently reported as immediate concerns. Visible bruising and injection-related challenges were more prominent than fear of thromboembolism—an unexpected finding given that preventing major events is the primary focus of clinical guidelines.

## 4 Discussion

This qualitative study highlights the multifaceted experience of patients undergoing perioperative anticoagulation interruption and underscores the need for patient-centered strategies to optimize outcomes and reduce care fragmentation. Across 7 focus groups, we identified 5 core thematic domains that reflect patient-important outcomes beyond the major bleeding and thrombotic events that typically anchor clinical trials in this area.

From a value-based healthcare perspective—defined as the health outcomes achieved per dollar spent [6]—these findings point to specific opportunities to improve both outcomes and efficiency. Improving communication clarity enhances patient understanding, reduces anxiety, and likely decreases complications or unnecessary healthcare contact. Addressing system-level gaps—such as misaligned provider communication—could reduce patient burden and improve coordination. Patient narratives reinforce that value is not merely about clinical outcomes but also includes the process experience, which strongly influences engagement and trust.

Despite process differences between DOAC- and warfarin-treated patients, patient concerns were largely transferable across groups. DOAC-treated patients received postoperative injectable anticoagulation, and unexpectedly, both groups expressed injection-related complaints including pain and fear. Major outcomes such as overt bleeding or stroke were not frequent concerns; instead, visible bruising and injection use were the most prominent.

These findings align with and strengthen the rationale for anticoagulation stewardship [12,13]—a concept akin to antimicrobial stewardship, where the goal is to ensure anticoagulants are used appropriately, safely, and effectively across care transitions. The perioperative period represents a high-risk window for both thrombotic and bleeding complications, and the absence of standardized, coordinated protocols often leaves patients uncertain or overwhelmed. Our results are consistent with a prior anticoagulation-related systematic review including 9 qualitative and mixed-methods studies exploring physician and patient perspectives, which identified key shared themes including the need for clear information, risk-benefit weighing, and defined roles in decision making [14].

Our results suggest that stewardship programs should extend beyond prescribing algorithms to include communication strategies, patient education materials, coordination tools (eg, calendars, reminders), and access solutions (eg, telehealth for perioperative consultations). The results also can inform strategies to implement improved patient counseling during the perioperative period. Based upon participant feedback, patient counseling should include advising patients about the risk of side effects (eg, bruising, pain) at the injection site during enoxaparin use, instructions on enoxaparin injection administration, informational resources related to syringe disposal, and provision of information and instructions both verbally and in written form. A split subanalysis by enoxaparin usage was neither feasible nor anticipated in the primary aim. Management of expectations regarding the clinical relevance of small bruising versus major events may also be necessary during consultation. Providers should forewarn patients about expected injection-site bruising, reinforce injection technique at every encounter, review sharps disposal, and provide both verbal and written periprocedural instructions.

### Strengths and limitations

This study offers an in-depth patient perspective on a clinically important and underexplored topic. We included patients on chronic anticoagulation for both venous thromboembolism and AF, whereas most literature focuses on AF. Thematic saturation was achieved across groups, and findings provide actionable insights for anticoagulation stewardship and perioperative workflow optimization.

Limitations include a modest sample size drawn from a single healthcare system, which may limit generalizability. Thus, a potential limitation is the higher-than-average educational attainment of our sample, which reflects the sociodemographic profile of the northern Chicago metropolitan area communities served by the participating sites rather than systematic selection bias; education was neither an eligibility criterion nor an identified reason for declining participation. Generalizability to populations with lower educational attainment warrants confirmation in future studies. Voluntary participation may have introduced selection bias, with more engaged or resourceful patients choosing to participate. Participant reflections, while detailed and collected close to the interruption event, may have been influenced by recall bias.

### Future directions

An actionable next step is the integration of patient-reported outcome measures into future perioperative anticoagulation trials and quality improvement initiatives. Patient-reported outcome measures would systematically capture domains such as anxiety, confidence in instructions, treatment burden, and satisfaction with care coordination—metrics critical to understanding real-world success but often absent from traditional efficacy-focused studies.
