# Supplementary material for: Navigating the pause—patient experiences with perioperative anticoagulation interruption: a qualitative study
Source: Res Pract Thromb Haemost. 2026 Jul 28;10(5):106890. doi: 10.1016/j.rpth.2026.106890 (PMC13521166; doi:10.1016/j.rpth.2026.106890)
Supplement: Supplementary file 1 [file mmc1.docx]

**Central Illustration.- Navigating the Pause: Patient Experiences With Perioperative Anticoagulation Interruption**

Perioperative interruption of chronic anticoagulation as a vulnerable transition in care. Surrounding domains detected five core themes identified in seven focus groups

**Supplemental. Anchoring Questions:**

Emotional –

- Please describe your experience during the interruption of your blood thinner during the last procedure.

- What were your fears or concerns during the time you needed to stop blood thinners for a procedure?

- Can you share any specific concerns or anxieties you may have about the upcoming surgical procedure?

Physical

- What were your expectations regarding pain management during the period after surgery given use of bleed thinners?

- What role did blood thinners have during your physical recovery process after surgery, are there specific aspects you would like to discuss or plan for future interventions?

- In your own words, can you describe what you believe are the most important factors in ensuring a comfortable experience while managing perioperative blood thinners?

Lifestyle

- How do you feel about the information received regarding your blood thinner interruption, and were there any aspects that you wanted clarification on?

- Are there any lifestyle or personal considerations that you feel are important for the medical team to be aware of during your perioperative care?

- How would you score your participation in the decision-making process regarding your anticoagulation interruption, were there specific aspects you feel particularly strongly about?

**Supplemental** additional Representative quotations from participants by theme

| Theme | Selected Quotations |
| --- | --- |
| Value of Written Instructions | *"Well, I like the [written] package as a follow-up, but I, you know, like, I’m not a big texter, I’d rather talk to somebody in person. I think you get just better information, and, you know, the like face-to-face meetings to me are always the best." (004, DOAC)*  *"Here's what we talked about. Here it is in writing so you have it and you can refer back to it and know exactly what the process is." (012, DOAC)*  *"I prefer verbal communication at all times, even on a phone. And I like a note summary follow-up. I think the verbal communication is very important." (082, warfarin)*  *"I prefer the, from the doctor, physicians, whoever, explain it verbally to me face to face, then I get the notes that I can reference, because if I don't remember something or I get something mixed up, the notes are right there to just flash it back to my head." (083, warfarin)*  *"I like the verbal communication, whether it's face to face or just hearing your voice reassures me. So I prefer, you know, I like the writing afterwards so I could summarize it." (084, warfarin)*  *"My clear preference, I want to see the writing afterwards because I keep a log of all my medical stuff. But what I wanted was your voice. And I could tell from your voice if you're anxious or not, you never were. So verbal communication worked for me." (084, warfarin)*  *"And it's usually communicated to me via [EHR portal name redacted], which is a good, good system, but I'm not crazy about it, because I got to sign in, go in and read. And I know you guys don't send it to my personal email, which would be great because I can easily print it out and transfer it onto my calendar." (087, warfarin)* |
| Injections | *"There was never any instruction given on how to do a subcutaneous injection. So, it was just, I don't know if they just missed it or if it was assumed since I had it, you know, had done it before but I kind of you know that was a few years before. And so I had to kind of read, like, try and remember exactly. Okay, you know, you do it in your stomach and you do you switch sides, you know, every day otherwise it gets too sore. And so, that really was the only thing I think that they missed." (004, DOAC)*  *“…I didn't know how to do, I didn't know how to do the shots myself, I did have somebody else to do them because I couldn't do it myself, but that's the only issue." (007, DOAC)*  *“…watching the video and watching exactly how to administer the [enoxaparin] Lovenox® and stuff like that. You know, the proper way to do it, where to do it. So the video after the tele-visit was very helpful also." (011, DOAC)*  *“And yeah, so I guess getting used to giving yourself an injection would be, you know, a warning for them and get someone to show you how to do it also. Right. That's also important. My sister-in-law is a nurse and I had her come over one day and say, “show me how to give myself this needle.” And she did. So that was very helpful." (088, warfarin)*  *"I hated the [enoxaparin] Lovenox®, I didn't like injecting myself." (134, warfarin)*  *"20 minutes of burn." (133, warfarin)*  *“…but what is obviously the fear of the needle itself, but two was the pain I experienced right after the shot. I had, I mean, the second I went in, I had pain right away. So I couldn't get myself to get over that mental part of doing it myself. So I had my husband do it...Nobody told me that there'd be pain like I had, yeah." (007, DOAC)*  *“I would say the fear kind of goes away after a couple times, but I still did not like it. There was a dread there every time I had to do it." (009, DOAC)*  *“…but you know, the alternative is, you know, what the alternative is I could have died. So you just grin and bear with it and do it but injecting yourself every day is not the most fun experience." (086, warfarin)*  *"Yeah, the proper disposal. There was no like, if I remember like one of my knee surgeries, they gave me a biohazard plastic container or whatever to put the hypodermics. But this time, so I had seven hypodermics and there was nowhere to discard it. So I just put the cover back on and threw it in garbage." (004, DOAC)*  *" But it's just what do you do with the needles? And I think if I remember correctly, I collected them and I did end up bringing them I think to the hospital here to disposal of them. I didn't know what to do. I didn't, I wouldn't throw them in the garbage because that's no..." (005, DOAC)*  *"The only issue I have in the whole thing is disposal of needles. I have a lot of them. I bought from [online retailer name redacted] a special container, but I've had great difficulty getting rid of them and I'd love some directions on how. I've called the fire department, I've called the police department, I've called, I've gone to [retail pharmacy name redacted], I got balled out for doing that. I went to the surgeon’s office they said “we don’t take them.” So I've tried five times to get rid of the needles and I don't have a clue what to do. So the only direction I need and haven't received is how to dispose of needles." (084, warfarin)* |
| Access to Team | *"But I'd have a virtual appointment with my vascular doctor, and that worked out well." (001, warfarin)*  *"I think I said I'm going to have surgery (chuckles,) they found a way to fit me in pretty quickly. So if there wasn't anything time sensitive, then it's probably more of a challenge, right?" (002, DOAC)*  *"Very easy to call the office to talk to is the nurse that handled everything and his office assistant. And it was, I was very, very impressed." (005, DOAC) [Context: also dealing with COVID and MRSA]*  *"It was a little difficult because we had to coordinate with several doctors. So I had to coordinate with my primary doctor, with the vascular surgeon, and with the hematologist... I knew when I was supposed to stop taking the pills. My, my, my problem was, I wasn't sure when I was going to be admitted. And how the heparin drip was going to start once I was admitted. That was a little frustrating and a little nerve wracking." (009, DOAC)*  *"...not enough to reassure me that everyone knew what was going on and that everyone was on the same page...It was difficult. So I would if I, if I spoke to the doctors, if I when I spoke to my surgeon, he'd say, Okay, we're gonna, we're gonna, we're gonna take care of this. I'll talk to the vascular surgeon, and we'll take care of it. Okay, but you only speak to the doctor when you have an appointment. If I did not have an appointment with the doctor, I was dealing with their staff. Staff did not always know what was going on." (009, DOAC)*  *"Because our, I'm in UW, Swedes in Rockford, and they didn't have anybody that could do my procedure. So I had to go to [Hospital] or whatever, [Hospital System]. We had two hospitals. We had to drive in like 70 miles to come in for it. So it wasn't real convenient with that." (083, warfarin)* |
| Clotting Risk | *"My only concerns was being off the [Apixaban] Eliquis®, how soon a blood clot could form since I had two pulmonary embolisms. That was my only concern." (002, DOAC)*  *"How long does it take for there to be a danger of clotting if you're off for four days, three days?" (003, DOAC)*  *"And so every day I'm worried that, you know, with the medication, without medication, I'm worried there's a blood clot." (012, DOAC)*  *"Just as to a little worry and fear, on my part, the blood clotting." (082, warfarin)*  *"When I was first going through, I was always worried about clotting, but the follow-up with getting your INR tested makes you feel like you got some kind of, if something's wrong, you're gonna kind of catch it and adjust to your dosage and stuff that needs to be done to stay healthy." (010, warfarin)*  *"Um, I had no fear of clots. I've followed the, my medical instructions as well as I can and take them very seriously. So I avoid those complications." (083, warfarin)*  *"On the other hand, I talked to Dr. [name redacted] at length, and he also talked at length with my surgeons. And Dr. [name redacted] said to me, “Look, there is a risk that you could get an embolism. We will deal with it.” And I just have such complete faith on Dr. [name redacted], and I had extraordinarily good surgeons who were very thorough. So I just said, if Dr. [name redacted] said he could manage it, I believe him. And I really did not have anxiety about it." (084, warfarin)*  *"I didn't have a stroke. I don't have time to have stroke. So it does bother me a little bit that I'm unprotected a little bit. But I think the bridging helps, doesn't it?" (133, warfarin)*  *"I'd rather bleed than have a stroke." (133, warfarin)*  *"So yeah, I'm still wondering whether I'm going to have another bunch of little strokes or another big one. I don't think I'm going to have a big stroke, but I don't really want to have any kind at all." (135, warfarin)*  *"I think some of my concern comes from, I personally knew three people who did not survive PE. Two of them have been in surgery. So maybe I'm reacting, but...It’s on the back of my mind." (082, warfarin)* |
| Satisfaction with Communication | *"...I didn't have any issues with the communication part." (007, DOAC)*  *"Well, to tell you the truth, I guess I had no frustrations, because I seem to have gotten, I got direct instructions." (008, DOAC)*  *"Yeah, so mine went very smooth. I think less than a month before I had my surgery, I talked to the vascular doctor. And so he made an appointment with the physician's assistants for her to call me like a week before the surgery, just to go over the details on how to stop the medication and then how to start the [enoxaparin] Lovenox® medication after surgery." (011, DOAC)*  *" I've been pretty satisfied with how it's been explained to me. And then the follow up instructions on print, um, they've always been, I always had somebody accessible if there's any other questions. So at this point I have nothing I can say that I would look to to improve any of that..." (083, warfarin)*  *"We knew everybody communicated with each other, and it seemed to be a good team." (086, warfarin)* |
| Provider Trust | *"It really makes a difference to have good healthcare people." (083, warfarin)*  *"You know, I trust you guys 100%." (085, warfarin)*  *"The surgeon, but when I objected to not receiving the extra blood thinner, he said, well, that was the cardiologist's decision. And I didn't even know he was involved other than a pre-exam before the surgery." (082, warfarin)*  *"I just leave it up to my doctors and physicians. I figure the main thing is in kind of hitting around the room here, confidence in your caregivers is utmost important... I'm never scared because I go to the professionals, they tell me what I need to know. Everything's been great. I've never had a problem with a doctor. And so I'm never scared going into a procedure. I got the instructions, follow the instructions. If we do okay, 99% of the time we're going to come out of it okay. And so I just can't stress enough how that confidence, knowing your doctor, having good relationship with your doctor...So that to me is so important is communication, confidence in the people, get the people to trust and just let them do their thing. I would never go to a, I mean, I know about auto mechanics and things, but if I'm having a problem with my car engine, I'm not going to take it to a shop and tell them how to fix it. They should know how to fix it, leave it with them, let them do their thing and on you go. Same thing with a doctor. They're just working on me instead of my motor, that's all." (083, warfarin)*  *"You know, I think what I would recommend, one thing I would recommend for someone new, I find it very helpful to have the continuity of one person. And as I say, I have [name redacted]. And if, you know, I said to her last week, “oh, do I have to go to [clinical site name redacted]? Can't I see somebody else at [clinical site name redacted]?” And she said, no. She said, (laughing,) “no, I want to see you.” So I think seeing one person and being, you know, having the continuity of that is very beneficial." (088, warfarin)*  *"And my net word is gratitude. Confidence and gratitude." (084, warfarin)*  *"I had no issues. I follow their directions. Like she said, they are the experts. And I just follow what they said, what they tell me." (001, warfarin)*  *"I'm one of these patients that do what the doctor tells me to. I figure the doctor knows way (says “way” exaggerated) more than I do." (003, DOAC)*  *"Just follow the instructions what they tell you to do. That's all I do." (134, warfarin)* |
| Clear Scheduling Guidance | *"It was very specific... three days prior to the surgery, you know, I'm stopping these medications, two days, you know, so the last time I took an [Apixaban] Eliquis® was, I believe, a day before the surgery..." (004, DOAC)*  *"...it was very very detailed and I was I was very impressed with all the information and things that I didn't even recognize or know it explained and yeah so that that was very helpful for me and also you know as far as NSAIDs and this and that you shouldn't take this and this…" (005, DOAC)*  *"...I merely had to quit taking the medication on a particular day, and commence on a separate day, on a later day, yeah, it was a simple process for me, and the communication was straightforward." (012, DOAC)*  *"Um, it's just being aware of what's going on and getting good instructions from, from your physicians and doctors and things helps tremendously... So it was all very simple. I just had when to stop certain medications, I would do that. They told me that evening after my last operation, that the hospital would have all my prescriptions and I'd start right back on all my medications that same day. So I never had to worry about the timing of it. That was all taken care of through the care I was getting, which I very much appreciate." (083, warfarin)*  *"The only thing I would say is the cons about it is maybe with somebody that doesn't have the [healthcare] background like that, that I would have might be overwhelmed. If they may have found all this information a little, quite overwhelming and maybe it were like, where sometimes when you read all these things, it just gets to be too much. And then you forget things or you just, too much information is, it's just, you know what I mean? It just, I just would think that's the only problem." (005, DOAC)* |
| Tracking via Calendar Tools | *"I just wrote it down literally like a calendar and just had, you know, which which medication to take on which days." (006, DOAC)*  *"For myself, I just do it myself. I do it for myself. I just do it myself. Like I said, I bring the I tear the sheet out of the calendar. I bring it down on the kitchen table. And it's there every day. I don't move it. It stays there every day. And I know what to follow based on the instructions coming out of your office." (087, warfarin)*  *"...I would transfer that to my personal calendar so I'm not off track…I would get notifications that way so that I'm not off track. And the notifications are like reminders." (001, warfarin)*  *"Reminders, yeah. So I have a calendar book for reminders...It's a book that's on my desk at home. I refer to it every day." (082, warfarin)* |
| Bruising Awareness | *"...I had a lot of bruises on my stomach from the from injections and that…" (007, DOAC)*  *"Uh the only thing that was kind of alarming is that uh the bruising around the uh injection areas that's the only thing that was that's other than that I had no problems with anything." (010, warfarin)*  *"I didn't expect the bruising. So I wasn't warned of it as symptoms or something, or maybe I didn't, I glanced over that." (011, DOAC)*  *"Expect some kind of bruising. Some could be little, some could be a little bit bigger. Don't be too, don't be, don't be too alarmed..." (010, warfarin)*  *"...I get the patches of bruising and I got this big dog that catches me with his claws. And so I try to be more careful when it's like I knew I was off the warfarin." (083, warfarin)*  *"Occasionally, I get the black and blue marks, you know, but that's all part of it doesn't concern me." (003, DOAC)*  *"...my stomach was black and blue..." (086, warfarin)*  *" And my stomach turns black, blue, yellow, green, it's like a rainbow of colors." (087, warfarin)*  *"No bleeding, no just a lot of black and blue." (088, warfarin)* |
| Fear of Excessive Bleeding | *"Not really. I mean, I bled enough to become a little bit anemic, but that's typical." (082, warfarin)*  *"I fell…in the bathroom…I had uh, subdural hematoma, and then six weeks later, I fell again, and I had another subdural hematoma and that time I was at…maybe I was here…but anyway, some young doctor took me over to Evanston Hospital, ran this tube up my arm, shot some glue into the thing and stopped it from bleeding." (085, warfarin)*  *"My, my biggest thing is how long do you go before you say, Hey, I need to call the doctor cause I didn't know. And at that point, then I thought, well, maybe it's, I'm overthinking this and I shouldn't go, you know, I was double, double questioning whether I should go or not. And it turned out I should have went because, you know, I obviously could have been something even more." (007, DOAC)*  *"I went through two pairs of underwear and I'm like, this ain't normal at that point. You know what I mean? I was like, at first it was just a little bit and then all of a sudden I'm just leaking blood. Then I'm like, okay, something's wrong here. And you know, it was just from moving. So at the end of the day, I was like, I have to go get checked out. Otherwise I didn't know if I was going to keep bleeding internally or just externally...It was just the bleeding what to do in that situation. Other than that I think everything else kind of went smoothly I just I just didn't know what to do the bleeding part." (007, DOAC)*  *"And so I never had this. I'm looking for any excess blood coming from a procedure and never had it. But I was told that if it happens, call your doctor and go to the emergency room." (008, DOAC)*  *"So I try to be careful. I don't work on cars anymore, thank goodness, or I'd be cutting up myself every day (chuckles.).* *But just to be aware of it and keep bandages and things like this handy because I know a little nick, a bump, things like that can cause the bleeding. It just happens easy, but it's easy to stop the bleeding too." (083, warfarin)*  *"I'm careful enough and I don't know how I could put it, but just being more careful not to have a problem. So I haven't had any problems. Of course, when I'm off of the warfarin, I would suspect the bleeding wouldn't be such a problem, but that's when the clots, they worry about the clotting. So I have not had any problems in the transitions at all because just being aware, being careful, watching your step and keeping the dog off of me, things like this to avoid having that contact that would cause a problem." (083, warfarin)* |
| Reliance on Personal Tracking Tools | *"Yeah, the summary paper. I write everything down on that so that's my that's my form of information so it knows like if I did it that day I’ll scratch it off…" (007, DOAC)*  *"So after surgery, I had a pad of paper. And every time I took medication, I wrote it down. And I had when I when we started the surgical process, I was given a folder with papers and instruction. And I had my pad of paper, those those instructions in the folder. And they were next to my medications. And anytime I took a medication, I wrote it down and I wrote down. So I would have like, I would write down the day, Monday, and then Monday, the date, and then what time I took the medication. So I would have a list of every medication that I took, what time on Monday, and then I would quit Tuesday. And then I would write everything down." (009, DOAC)*  *"I'm very organized, and I took the [Apixaban] Eliquis® and I placed it up, you know, I have a, I take quite a few medications, like close to 20 a day, so I took that out of my stack of medications and I placed it up in the cabinet so that I wouldn't even be, you know, when I was going through to get my ninth med, I take it at bedtime, you know, to not be looking forward to that, that I just say, oh, that's right, I don't take it, you know. " (005, DOAC)* |
| Reliance on Partner | *"And also any instructions I got, I also gave it to my wife so that she would watch things as well." (008, DOAC)*  *" My husband would make sure that I was taking my meds on time." (009, DOAC)*  *"My wife likes to know about ‘em, but I pretty much manage them myself, but I'm not very organized and she is, and she's the techie of the family. So she takes care of that and she's always there for support or reference if I need it." (083, warfarin)*  *"And my wife is meticulous about keeping my, my pills, you know, so, I mean, as I've gotten older, I, my, my, uh, memory is starting to slip and, uh, you know, if, if, if, if I start making things both sideways, she'll step right in, you know...My wife manages every one of them [3 pillboxes]." (085, warfarin)*  *"My wife was aware of all of that stuff, and she read the stuff, too, to remind me to double check that I'm doing the stuff." (135, warfarin)* |
| Food Concerns | *"It is too bad, because you know, you try to stay between two and three and watch your greens and all that stuff. And, you know, when you when you're on track, and then you have to restart, then you're off track again. And it's just a pain in the butt." (087, warfarin)*  *"So I know with variations with my diet, if I'm a lot of doing rushing around or out of town, I don't eat as many vegetables as I normally would. And my blood dose out of zone is too thin from the dosage." (083, warfarin)*  *"...my experience with the greens initially, you know, I was very frustrated because I'm not a big greens eater. And the effect of greens on warfarin is amazing. And the nurses I dealt with, you know, always explain things very clearly to me. And they said, you know, you just eat your regular diet, and your greens every week, don't change, don't go up and down on your greens. Yeah. And she says, we'll make the necessary adjustment with the medication. If the greens don't work for you. But she said, maintain it every week." (087, warfarin)*  *"So I would love if you have any ideas or um, I don't know, even give me some tips in what I should be eating. I know that kale and spinach and all that has too much vitamin K, um, but then they say, but make sure you have your salads. So, you know, it's a little confusing." (088, warfarin)*  *"I guess maybe for me, I need a whole tutorial again, on diet, because I, it seems like no matter what I eat, my numbers are off. And I don't eat badly, you know, so I need to, I need to have a rehash about, you know, greens, greens, greens, greens. That's what I need to figure out." (088, warfarin)* |
| Perioperative Bleeding Fear | *"Because before I would have to, uh, go off the coumadin for like…five days. And then I'd have to go back on right after the procedure. And there was a fear there." (001, warfarin)*  *"And they started the warfarin, but it took almost three weeks for my INR to get back to where it should. And they kept increasing my warfarin." (082, warfarin)*  *"It took me four or five days to transition back to get my initial scores were low, where I was more like 1 at 1.3. Dr. [name redacted] raised my [enoxaparin] Lovenox®, I may be pronouncing that right wrong, from 40 milligrams to 100. And two shots of that over two days did the trick. And I was right back where I needed to be." (084, warfarin)*  *"I had three or four people watching my INR at the same time, so I had a lot of communication, and we stayed within the range." (086, warfarin)*  *"When you’re out of whack, then you're in trouble. So it's you need a lot of communication in the beginning of startup. Once you reach a level then you know it's not quite as much communication as needed." (086, warfarin)*  *" You know, like, interruption works, but the bottom line is it's not necessarily the interruption, but it is a specific aspect of restarting...Recovery from the interruption. Yeah. Back on the getting back on the bus." (087, warfarin)*  *"See, I haven't had much trouble with the interruptions, but I have more trouble the regular period when I can't get it back, you know, between two and three." (088, warfarin)*  *"You have the procedure, then you have to go back, start Warfarin, keep [enoxaparin] Lovenox going, go to INR clinic, make sure you're between two and three. Once I was 1.9, 1.8, so I got to come back, get a check. Another time I was 3.3, then they wanted me to go in for a blood test to verify the INR number." (087, warfarin)*  *"But the thought I had was, it would be nice if we can purchase, whether it's from North Shore, or on our own, I know they're kind of expensive, if we can purchase a meter and check our own INR, and then go to the clinic, you know, if it's like 4.0 or something or 1.8. Or if it's in range, then with that consistency, maybe just go to the clinic once every three months or four months or something. Now I, I know that means, you know, less visits and less billing and all that stuff. But this isn't about dollars and cents. This is about patients, and what's convenient and good for them. So I don't know how feasible that is about actually us having a meter and check our INR at home, whether it's once a day, twice a week, whatever the case may be, and then pop into the clinic, you know, whatever every three or four months, just to check." (087, warfarin)*  *"But the recovery you're getting back on the bus is the part usually that varies, because you can come back, and you can be below two. And then you got to come back, you can be within two and three, but you need two consecutive days. So you come back again, and you might be 3.3. Okay, so then you got to come back again and do the INR again. I mean, it's just, you know, it's like a teeter totter, back and forth, back and forth, back and forth." (087, warfarin)*  *"Yeah I don't know if concerning is the right word or if it's just a big annoyance because you're trying to get back into that two to three range so I don't look at it as a problem it's just an annoyance come on let's go let's get between two and three you know and how many times do I have to go to the clinic to you know to get that verified and back on track then you know." (087, warfarin)*  *" It's not that it's, it's not that it's difficult, but disruptive is a good word, you know, because you're running around back and forth back and forth back and forth." (087, warfarin)*  *" I really don't have any fear. I mean, I know what the procedure is. I know what it has to entail. And because I'm pretty much on track, most of the time going back onto it is not that hard. I know that I bridge for three or four days and take my dosage of warfarin, and within a week I'm back up to normal levels. So I really, I'm not worried when I go off of it." (086, warfarin)*  *" How do I cope with it? I just, you know, I just cope with it. I just look at it as this is what I have to do, and this is what I have to take, the [enoxaparin] Lovenox® and reintroduction of the warfarin. There's not much I can do from a coping standpoint. I may be concerned, you know, about, you know, whether I'm properly protected or not, but I, you know, as a patient, there's not much I can do. I just put my faith and trust in the instructions that come out of your office, and I work with it, even though it's a pain in the butt, but, you know, I do it, you know, that's about it." (087, warfarin)* |
| Access Delay | *"It's a little bit of a headache having to physically go in and see each of those doctors and get scheduled to see them." (002, DOAC)*  *"And so if the surgeon who in my case was Dr. [name redacted], if his office would take care of scheduling with the anticoagulant doctor and scheduling with your primary care physician, instead of making that the responsibility of the patient, I think that would make sure that you've got all your bases covered and not have to try and go through the system, which sometimes works and sometimes doesn't work." (004, DOAC)*  *" In fact, that's one thing I think could be corrected. If you have a patient that's 3.3 or above an INR, and the nurses want you to go in for a blood test to verify or confirm the reading, that patient should have immediate access to the lab to have the blood drawn and not sit there and wait for two hours till an opening occurs." (087, warfarin)* |
| Confidence on Anticoagulation | *"No, no, it's it, as I said, it's the fact that this whole thing is invisible to me. Right? And you guys have all kinds of things you can use to look and see and test, but it's invisible to me. And yeah, at the end of the day, I just have to have faith that the medication is doing what it's supposed to do. Because I yeah, it's not like, you know, an injury that you can see and you can monitor it's just in your body." (012, DOAC)*  *"So Warfarin is a good drug, a powerful drug, I understand it..." (087, warfarin)*  *"...with the hip replacement, they didn't give me anything but the warfarin. And that really, really concerned me. And I was asking a surgeon and my cardiologist about that. And they said, “Well, we don't feel it’s necessary. They have to weigh the risk between a clot and a bleed.” And I said, “Well, you can stop a bleed, but you can't always stop a clot.” I don't know how true that is." (082, warfarin)*  *"You know, one of the things that always bothers me when I follow your protocol is, am I properly protected? Because I know Warfarin is a good medicine. But, you know, when I'm off of it, and I got to take [enoxaparin] Lovenox® for in this whole five-day period, generally, I'm always concerned, you know, am I properly protected or not? And it's always in the back of my mind. But I figured you guys know what you're doing. So, you know, I follow the procedure." (087, warfarin)* |
| Medication Contrast with Warfarin | *"I was on [Apixaban] Eliquis® at first, but then they said, well, [Apixaban] Eliquis® is not helping me as far as thinning the blood and for my system to actually absorb the medication. I had gastric bypass surgery years ago, but there is a malabsorption that they had to consider. And so that's why I'm on Warfarin. It's a harsher drug, but my body absorbs it better." (001, warfarin)*  *"Well, [Apixaban] Eliquis® was just an easier drug to deal with, whereas with the warfarin, I have to go into the clinic, the Coumadin clinic more often. And it's basically because I've never really been in the therapeutic range that long because of the fact that my system does not…um…well my system does not absorb that well." (001, warfarin)*  *" When I got out of there clean, they put me on equinox. [Subject mistaking the name of Eliquis® [Apixaban]] I started out on equinox[Apixaban] [Eliquis®], and then I met Dr. [name redacted]. He did some testing on me, and they found that the equinox [Apixaban] [Eliquis®] was not working, and I was getting a repeat of the embolisms. So what happened next is I did some genetic testing and discovered I have a genetic predisposition to cause embolisms. Dr. [name redacted] switched me to warfarin, and I've been pretty good ever since." (084, warfarin)*  *"...the price of [Apixaban] Eliquis® is so expensive and they are just, you know, and that's, I know I was on Coumadin for a while and then now the last time I've been in my donut hole, the last time I'm now getting it at a zero copay as a hardship because I'm only on social security, but, um, my last time they wanted almost $500 a month for my Eliquis® and that's the only thing when it gets to be like that. I mean, what do you do?" (005, DOAC)*  *"I was on Aspirin for years. I had had experience with blood clots 20 years ago, maybe a little bit more. And after that I was on Aspirin. I'd been on Aspirin for years...And then after the [Apixaban] Eliquis®, they adjusted my medicine and I'm now on [Clopidogrel] Plavix and [Rivaroxaban] Xarelto®." (009, DOAC)* |
| Medication Cross-Check Reassurance | *"She was the nurse and she would um…confirm, confirm, first of all, she would go over all my medications that I'm taking…She'd tell me the exact date to stop and the exact date to start, to stop, wait, the exact date to stop taking the coumadin and the exact date to go back on." (001, warfarin)*  *"...two calls preoperative from the hospital. The first call was a week before my surgery, and she went through every one of my medications and when I should continue or discontinue those. And then the person that called the day before the surgery went through it again. So I felt very comfortable that we were all on the same page." (006, DOAC)* |
| Technology Tools | *"I have Echo Dot reminding me when to start it and when to stop it. " (001, warfarin)*  *"...on my iPhone calendar stating, you know, Tuesday night, stop medication." (011, DOAC)*  *"I use Apple, put it in Apple Notes, it appears on all my devices, whether it's computer, iPad, iPhone, and then I entered on my calendar."(084, warfarin)*  *"Alexa tells me every night to take a pill because I can't remember to do it." (133, warfarin)*  *"Yeah, I like the [patient-facing portal name redacted]." (135, warfarin)*  *"I am teaching myself AI and I use it to ask questions. I use the chat GPT or else I use co-pilot and I will ask questions and then I will go and verify it because it will always give a couple sites and then I'll go because I also use it on myself because I've gone through cancer... It helps me to understand stuff in a simpler term but my doctors have always dumbed it down for me too so I never have have uh major questions for my own personal health." (wife of 083, warfarin)*  *"I do copy and paste like when I would get results from our tests and we don't want to wait for the doctor from from my chart I'll copy and paste what the results are from the test and I'll put it in AI." (wife of 083, warfarin)*  *"And I will often run it through three different AI platforms, just to check. I don't just use chat GPT, I use Claude, I use Gemini. And I just take the same I just take the report that I get from [patient-facing portal name redacted], I drop it in, I said, all I say is explain it to me like I'm a 15-year-old, or a 10-year-old or whatever. And then I compare the answers from multiple platforms, because I don't trust- I trust you a lot more than the platforms. And I get a consensus of what it is. And then if I have any remaining questions, I'll call you back." (084, warfarin)* |
